# The Book World of Medicine and Science

**Published:** 1898-03-19

**Authors:** 


### THE BOOK WORLD OF MEDICINE AND SCIENCE.

The Treatment of Imbeciles and Epileptics. By Dr. Rhodes and Alderman Macdougall. (Pp 36. Illustrated. Manchester. 1897. *Poor Law Officers' Journal*, Queen Street.)

This volume consists of a report by the authors—who were commissioned by the Chorlton and Manchester Asylum Committee—on certain institutions in Germany, France, and Belgium for the treatment of imbeciles and epileptics. It is well and copiously illustrated by maps, plans, and views. The institutions reported on are chiefly those managed on the pavilion or colony plans. In the pavilion plan arrangement is made for the maintenance in one building of about 30 patients; in the colony plan provision is made in small villas for only a few patients, and each villa is under the charge of a House Mother and Father. Messrs. Rhodes and Macdougall have come to the conclusion that the former plan is the more suitable for patients who are physically disabled, and that the latter allows those who are more self-reliant better to follow up individual pursuits. The report is clearly written, and its conclusions will, we think, be endorsed by most who have paid attention to the subject. For years those who have inveighed against the barrack system—whether for pauper children or lunatics—have been as voices crying in the wilderness, but at last there are signs that this country is realising the value of Continental methods.

The Medical Annual. 1898. Sixteenth Year. (Bristol: John Wright and Co. Pp. 700. Price 7s. 6d. net.)

We are glad to welcome the issue of another number of this admirable annual. To those who read the medical papers as they come out, their number is so great and their contents so enormous, that it is a comfort to have such a resumé of progress as is here given, well bound and indexed and easy to refer to; while to those who read but little it is perhaps even still more important to have at hand the yearly volumes of a work which is becoming more and more the general practitioner's companion and handy work of reference. Whether a question refers to medicine or surgery, to midwifery or to sanitary science, if it is anything on which new discoveries have been made during the year, one is almost sure to find in this volume some record of what has been done, or some reference to larger works where further information may be obtained. Many of the illustrations are very good, and we would specially draw attention to the coloured plates illustrating Mr. Samuel Shattuck's article, entitled, "An Atlas of the Bacteria Pathogenic in the Human Subject." The book also contains a great deal of general information on subjects connected, not with the science, but with the daily work of our profession, and altogether it will be found a very useful handbook.

The Tallerman Treatment by Superheated Dry Air. Case Notes and Medical Reports, with numerous Illustrations. Edited by Arthur Shadwell, M.A., M.B.Oxon., M.R.C.P. (London: Baillière, Tindall, and Cox. 1898.)

This is a book which should be read by all medical men who wish to inform themselves as to what can be done by a method which is somewhat outside the ordinary medical routine for the relief of rheumatism, gout, rheumatoid arthritis, and other affections of joints and fascia attended with pain and stiffness. The Tallerman treatment is described as a new method of applying heat locally to the cure of disease and the relief of pain, its most important feature being the use of dry air, which allows a much higher temperature to be applied to the part affected than would otherwise be possible. Hot water, as is well known, becomes painful at about 115 deg. Fahr., and vapour or steam cannot be borne above 120 deg. Fahr., whereas it has been ascertained that hot air, when dry, can be tolerated up to 300 deg. Fahr., and even higher. The book contains many careful observations made by Dr. Sibley in regard to the effect of the local application of hot air according to this method on the general temperature, the pulse, and the respiration. The main part of the volume consists of records, in many instances admirably illustrated, of cases which have derived benefit from this treatment. The cases dealt with are principally rheumatic arthritis, rheumatism, stiff joints, sciatica, and sprains; but an interesting paper by Dr. Walsham is included, giving his experience of the method in the treatment of flat-foot. It is impossible to peruse this book without seeing how powerful a means of treating a most obstinate class of diseases is placed at our disposal. Altogether, it is an interesting clinical record.

Premature Burial: Fact or Fiction. By David Walsh, M.D.Ed. (London: Baillière, Tindall, and Cox. 1897; pp. 49. Price 1s. 6d.)

Dr. Walsh has, under the above title, issued a vigorous counterblast to Messrs. Tebb and Vollum's horrifying pamphlet. Dr. Walsh reviews the circumstances under which burial takes place in this country; touches on the recognised signs of death, and forcibly demonstrates the utter inadequacy of the evidence on which Mr. Tebb bases his statements. The conclusions at which Dr. Walsh arrives, namely, that premature burial rarely if ever occurs where ordinary care is taken, and that the mass of reported "cases" are little better than legends, can hardly be seriously controverted. This little book will no doubt do good service in reassuring the timorous. No doubt there is no single infallible test of death except the presence of putrefaction, but at the same time it is quite certain that a medical man is quite competent, with a little care, to arrive at a correct conclusion in every case. It is singular how seldom medical men are asked to determine whether or no death has occurred. If the law required—as it should—that death certificates be signed only after inspection of the body, there would no longer be even the remotest probability of premature burial occurring.

### BOOKS RECEIVED.

Swan Sonnenschein and Co.

"Epidemic Diphtheria." By Arthur Newsholme, M.D., M.R.C.P.

Sampson Low, Marston, and Co.

"A Manual of Static Electricity." By S. H. Morrell, M.D.
"Twentieth Century Practice." Edited by Thomas L. Stedman, M.D.
"The Care of the Sick." By Dr. T. Bellroth. Translated by J. Bentall Endean.

James Willing Jun. (Limited).

"Willing's Press Guide, 1898."

John Wright and Co.

"The Medical Annual, 1898."

